# Alpha-ketoglutarate supplementation and BiologicaL agE in middle-aged adults (ABLE)—intervention study protocol

**DOI:** 10.1007/s11357-023-00813-6

**Published:** 2023-05-23

**Authors:** Elena Sandalova, Jorming Goh, Zi Xiang Lim, Zhi Meng Lim, Diogo Barardo, Rajkumar Dorajoo, Brian K. Kennedy, Andrea B. Maier

**Affiliations:** 1 Healthy Longevity Translational Research Programme, Yong Loo Lin School of Medicine, National University of Singapore (NUS), Singapore, 117456 Singapore; 2 Centre for Healthy Longevity, National University Health System (NUHS), Singapore, Singapore; 3 Department of Physiology, Yong Loo Lin School of Medicine, National University of Singapore (NUS), Singapore, Singapore; 4 Genome Institute of Singapore, Agency for Science, Technology and Research, Singapore, Singapore; 5 Health Services and Systems Research, Duke-NUS Medical School, Singapore, Singapore; 6 Department of Human Movement Sciences, @AgeAmsterdam, Faculty of Behavioural and Movement Sciences, Vrije Universiteit Amsterdam, Amsterdam Movement Sciences, Amsterdam, Netherlands

**Keywords:** Alpha-ketoglutarate, DNA methylation age, Biological age, Geroscience, Geroprotective intervention study

## Abstract

Targeting molecular processes of aging will enable people to live healthier and longer lives by preventing age-related diseases. Geroprotectors are compounds with the potential to increase healthspan and lifespan. Even though many of them have been tested in animal models, the translation to humans is limited. Alpha-Ketoglutarate (AKG) has been studied widely in model animals, but there are few studies testing its geroprotective properties in humans. ABLE is a double blinded placebo-controlled randomized trial (RCT) of 1 g sustained release Ca-AKG versus placebo for 6 months of intervention and 3 months follow up including 120 40–60-year-old healthy individuals with a higher DNA methylation age compared to their chronological age. The primary outcome is the decrease in DNA methylation age from baseline to the end of the intervention. A total of 120 participants will be randomized to receive either sustained release Ca-AKG or placebo. Secondary outcomes include changes in the inflammatory and metabolic parameters in blood, handgrip strength and leg extension strength, arterial stiffness, skin autofluorescence, and aerobic capacity from baseline to 3 months, 6 months, and 9 months. This study will recruit middle-aged participants with an older DNA methylation age compared to their chronological age, and test whether supplementation with Ca-AKG can reduce DNA methylation age. This study is unique in its inclusion of biologically older participants.

**Supplementary Information:**

The online version contains supplementary material available at 10.1007/s11357-023-00813-6.

## Introduction

Chronological age is the primary risk factor driving major chronic diseases, including cardiovascular disease (CVD), cancer, neurodegeneration, type 2 diabetes, sarcopenia, and osteoporosis. Geroscience research aims to understand the molecular and cellular mechanisms of aging as the main driver of chronic diseases and it has identified key biological tenets of aging, which, when targeted with drugs or supplements, result in the increased healthspan, compression of morbidity in aged animals and in some cases, the extension of lifespan [1–3]. Translation to clinical research in humans is still in its early stage. Accelerated aging results in reduced or impaired function of multiple organ systems, including the cardiorespiratory, metabolic, immune, and musculoskeletal systems. Age-related changes can be measured at the molecular, cellular levels and used as a guide for clinical interventions as the inclusion criteria and/or assessing the effectiveness of the intervention [3]**.** Among features of aging, the epigenetic modifications, changes in the genome that are not due to changes in the genetic sequence, are implicated in aging. These modifications commonly involve DNA methylation and histone modifications (including methylation and acetylation), which alter the accessibility of DNA and affect many nuclear processes including transcription [4]. Epigenetic modifications change with chronological age, responding to the environmental factors, indicating a degree of plasticity [4]. In human studies, the epigenetic load is quantifiable and can be a useful marker for measuring biological aging.

Alpha-ketoglutarate (AKG) is an intermediate of the Krebs cycle, which is involved in various metabolic and cellular pathways as a signalling molecule, energy source, and precursor of amino acid synthesis [5]. AKG is a weak acid containing two carboxyl groups and a ketone group. As a metabolite, AKG, is an antioxidant, regulates nitrogen and ammonia balance, as well as epigenetic and immune processes [6]. AKG is also a substrate for Ten-Eleven Translocation (TET) methylcytosine dioxygenases, which contribute to DNA demethylation [7]. Mitochondrial function decreases with age, which could contribute to the overall decrease in levels of AKG [8]. Several metabolites such as fatty acids, vitamins, microelements, nucleic acids, and amino acids occur naturally within cells [9, 10]. These metabolites have been identified to modulate aging, as well as exert organ-specific effects [9, 10]. Among these metabolites, AKG has been of a particular interest due to its essential role in cellular metabolism, energy production and aging. In this regard, AKG has been studied as a geroprotective agent in model animals [9, 11, 12]; in humans, the effects of AKG were tested mostly in haemodialysis and burn patients [13–20], only one retrospective study have assessed the geroprotective potential of AKG in a general population [21]. AKG can be administered alone or as salt with calcium, sodium, or ornithine [22]. Several clinical trials that used Ca-AKG in hemodialysis patients found it to be safe at doses up to 4.5 g/day, to increase plasma arginine and decrease plasma urea levels [17–20]. In post-menopausal women 6 g/day of Ca-AKG for 6 months was found to be safe and induced beneficial changes in serum levels of the bone resorption marker CTX (C-telopeptide type I collagen) consistent with the preservation of bone mass in the lumbar spine [23]. AKG reduced ischemic markers in circulation of men undergoing coronary surgery when added to blood cardioplegia for intermittent antegrade intracoronary perfusion [24]. AKG also prevented muscle atrophy in a Duchenne muscular dystrophy mouse model [25]. Thus, it appears that AKG has pleiotropic function and can affect multiple physiological systems [22]. Secondary outcomes in current study will aim to assess the effect of Ca-AKG on muscle health by functional tests (handgrip strength and 8 repetition maximum test), cardiovascular function by pulse wave velocity analysis, body composition and bone density by dual energy x-ray absorptiometry, aerobic capacity with cardio-pulmonary exercise test. Blood samples will be collected to measure inflammatory parameters, kidney function, fasting triglycerides, high-density lipoprotein, glucose, and liver profile.

In addition, the effect of Ca-AKG on the relationships of clinical and biological parameters to DNA methylation as well as a detailed profile of responders and non-responders can be studied in the post hoc analysis.

This study includes healthy participants with a higher DNA methylation age and will test whether 1 g of Ca-AKG can reduce this age.

## Methods

### Study design

This single-center, randomized, parallel, double blinded, placebo-controlled trial will investigate if 1 g/day of sustained release Ca-AKG compared to placebo can lower DNA methylation age in 120 middle-aged healthy individuals. Participants whose DNA methylation age is greater than their chronological age will be eligible for intervention.

All the participants will provide informed and written consent before the screening visit in accordance with the Declaration of Helsinki. The study has been approved by NUS IRB (NUS-IRB-2021-946) and registered at clinicaltrial.gov (NCT05706389).

### Intervention

The study is using 1 g/day of Ca-AKG sustained-release tablet (Ponce De Leon Health, USA). The placebo ingredients are listed in Supplementary Table 5. The Ca-AKG and placebo pills and bottles are identical in appearance.

### Pre-screening and screening

**Fig. 1:**
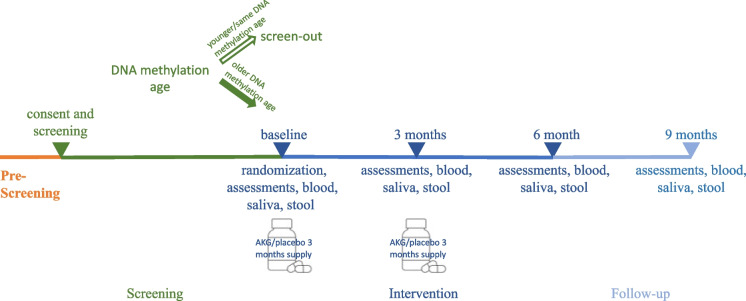
ABLE study schedule. Pre-screening of interested individuals is done by email or phone. At screening consent is taken followed by blood sample for checking the screening criteria. Eligible participants are invited for baseline visit when the randomization happens. They will come back at 3 months, 6 months, and 9 months to complete all the assessments

Potential participants who express interest will be pre-screened by email or phone call (Fig. 1), when their chronological age and health status will be confirmed. If they fit the inclusion and pre-screening exclusion criteria (Table 1), they will be invited for a screening visit. At the screening visit after signing the informed consent, the fasting blood sample will be taken to assess their DNA methylation profile, fasting triglycerides, high-density lipoprotein, glucose, and liver profile (total protein, albumin, globulin, bilirubin, alkaline phosphatase, aspartate aminotransferase). The screening will include as many participants as necessary to achieve 120 randomized participants.

**Table 1:** Inclusion and exclusion criteria

Inclusion criteria	Pre-screening exclusion criteria *any condition*	Screening exclusion criteria > *1 condition*
40–60 years of age	Any cardiovascular disease, severe / uncontrolled hypertension, rheumatic heart disease, congenital heart disease, deep vein thrombosis, pulmonary embolism	Waist circumference ≥ 90 cm (males) ≥ 80 cm (females)
DNA methylation age > chronological age	Type 1 / 2 diabetes (oral metformin or insulin therapy), diabetic complications	Fasting triglycerides ≥ 1.7 mmol/l
	Active cancer or on treatment in the last 3 years	High-density lipoprotein < 1.0 mmol/l (males); < 1.3 mmol/l (females)
	Chronic obstructive pulmonary disease (COPD), severe asthma (daily medications)	Blood pressure ≥ 130/85 mmHg or use of antihypertensive medication
	Multiple sclerosis, autoimmune/immune deficiency diseases	Fasting glucose ≥ 6.0 mmol/l
	Recent history of sepsis or infection (hospitalization in last 3 months)	Osteopenia
	Any psychiatric disease or neurodegenerative diseases	Mild Osteoarthritis not interfering in daily activities
	Any metal implants in the body	Fatty liver
	Hepatitis / Liver cirrhosis	
	Severe kidney disease (GFR < 30 ml/min/1.73 m^2^)	
	Skin disease (oral medication)	
	On other investigational product within 60 days or 5 half-lives before the screening	
	Any serious medical illness (PI’s judgment)	
	Pregnant women or planning pregnancy in the next 9-months	

### Study visits

Eligible participants will be invited to the baseline visit when they will be randomized to receive Ca-AKG or placebo. At baseline, participants will complete the assessments, including a blood draw, saliva sample collection, anthropometry and physical assessments, carotid-femoral pulse wave velocity, body composition with dual energy X-ray absorptiometry (DXA), cardio-pulmonary exercise test (CPET), skin autofluorescence assessment and answer questionnaires including a socio-demographic survey, sleep and quality of life questionnaires, international physical activity questionnaire, global preferences survey and Montreal Cognitive Assessment test and menopause questionnaire (females only) (Table 2). Participants will receive their 3-months’ supply of Ca-AKG or placebo supplements, activity tracker (ActiGraph, wGT3X-BT), 3-day food record, physical activity diary and stool collection kit (AMILI Pte. Ltd.).

**Table 2:** Overview of assessments and visits

Methodology	Screening	Visit 1	Visit 2	Visit 3	Visit 4
Signed informed consent	√				
Screening Checklist	√				
Clinical assessments					
Anthropometry	√	√	√	√	√
Body Composition (DXA scan)		√		√	√
Arterial Stiffness (carotid-femoral pulse wave velocity)		√	√	√	√
Cardiopulmonary exercise Test		√		√	√
Muscle Strength Tests		√		√	√
Skin Autofluorescence test		√	√	√	√
Questionnaires					
Socio-demographic Survey		√	√	√	√
Sleep Questionnaire		√	√	√	√
SF-36 Questionnaire		√	√	√	√
EuroQoL-5D-5L		√	√	√	√
MoCA		√	√	√	√
IPAQ		√	√	√	√
Menopause Questionnaire	√	√	√	√	√
3-day food diary		√	√	√	
Physical activity diary		√	√	√	√
Global Preferences Survey		√		√	
Blood parameters					
DNA methylation	√	√	√	√	√
Complete blood count	√	√	√	√	√
Fasting lipid panel	√	√	√	√	√
Fasting glucose	√	√	√	√	√
Fasting haematological panel	√	√	√	√	√
Fasting protein panel	√	√	√	√	√
Renal Function test	√	√	√	√	√
Insulin & HbA1C		√	√	√	√
Metabolite concentrations		√	√	√	√
Inflammatory markers		√	√	√	√
Other samples and assessments					
Saliva Samples		√	√	√	√
Stool Samples		√	√	√	√
Activity Tracker		√	√	√	√

Three months, 6- and 9-months visits (Fig. 1) include assessments as described in Table 2. At 3 months visit the participants will bring back the bottles (empty or with unused supplements) and will also receive the new 3-months’ supply of Ca-AKG or placebo.

### Primary and secondary outcomes

The primary outcome in the study is the change in the median of four blood-based DNA methylation aging clocks, Hannum [26], Horvath [27], GrimAge [28], and PhenoAge [29].

Epigenetic age, or specifically DNA methylation age, estimated by aging clocks has previously been used as clinical trials outcomes. In a lifestyle-based intervention study, the intervention improved DNA methylation as measured by DNAmAge [30]. Another study showed that using a protocol intended to regenerate the thymus, DNA methylation age can be reversed measured by Hannum, Horvath, GrimAge and PhenoAge [31]. The median of these four clocks was able to detect DNA methylation age reversal three months earlier than the respective individual clocks, which is crucial for clinical trials of relatively short duration [31]. Therefore, DNA methylation age will be measured by this set of aging clocks as the primary outcome.

DNA from de-identified buffy coat samples will be extracted using QIAamp® DNA Mini kits. Quality of genomic DNA will be determined through A260/A280 and A260/A230 spectrophometry ratios and concentrations of DNA through the Qubit™ fluorometer. Approximately 1ug of genomic DNA from each study participant will be bisulphite treated using the Zymo EZ DNA Methylation Kit to convert non-methylated cytosine nucleotides to uracil for subsequent methylation profiling. The Illumina Infinium MethylationEPIC (EPIC) BeadChip (Illumina, USA) will be used for high-throughput measurement of DNA methylation on a genome-wide scale. The EPIC BeadChip enables reliable and reproducible evaluation of over 850,000 probes, covering 90% of the predecessor 450 K chip plus over an additional of 350,000 CpGs that offers improved genomic coverage of gene regulatory regions [32].

### Anthropometry, body composition, physical assessments, and activity tracking

Height will be measured with a stadiometer (SECA217, SECA Gmbh&Co KG, Germany) and weight will be measured with a floor weighing scale (SECA813, SECA Gmbh&Co KG, Germany). Body mass index (BMI) will be calculated. Waist and hip circumference will be measured three times with a cloth measuring tape (SECA201, SECA Gmbh&Co KG, Germany), at the level of the umbilicus (waist) and at the level of the symphysis pubis and the greatest protrusion of the gluteal muscle (hip). Body composition and hip and lumbar bone mineral density will be measured by the DXA machine (Horizon DXA system, Hologic, Inc., USA). Body composition is being expressed as body fat mass in % and kg, lean muscle mass in % and kg with the fast array mode as listed in Supplementary Table 1.

For the handgrip strength measurement, the participant will hold the dynamometer (Jamar Plus + , Sammons Preston Rolyon, USA) in a standing-handshake position [33]. The participant will squeeze the handle of the dynamometer with maximum effort. The test will be conducted three times on both arms, with 1 min rest between each attempt [34].

Participants will perform the 8-repetition maximal (8-RM) leg extension on a seated leg extension machine (640 LEC, GYMSPORTZ, Singapore), with the initial weight of 10 kg. If the participant is able to perform 8 repetitions, without being able to do the 9th repetition, the weight will be recorded as 8-RM strength. If the participant is able to do 9 repetitions for the given weight, the rate of perceived exertion (RPE) will be measured after every set, to determine the amount of next incremental load, to be added after every set, with a rest period of 5 min, until 8-RM is achieved (Supplementary Table 1). For a validated repetition, a complete range of motion has to be performed.

The participants will be issued an ActiGraph (wGT3X-BT device, CenterPoint software, ActiGraph LCC, USA), physical activity tracker, to monitor their routine activity for seven consecutive days after the visit, except while engaging with water activities [35]. It measures acceleration on three axes and can be used to estimate postures and energy expenditure derived from different types of activities. Raw acceleration data will be captured (Supplementary Table 1). Participants will also fill in the 3-day food diary [36]; their food intake on three non-consecutive days, one of which is a weekend day, will be recorded via an online form. Additionally, participants will record the time and duration when they removed the activity tracker via an online form. The tracker will be mailed back to the study team in a pre-paid envelope after completion of the 7-day activity monitoring. The physical activity tracking and food record will be collected after each visit for the purpose of monitoring if there are any changes in lifestyle.

### Cardiopulmonary exercise test

Participants will perform a graded maximal treadmill walking depending on their initial fitness level. The purpose of this assessment is to determine participant’s maximal aerobic capacity; thus, the test will be stopped when the volume of oxygen peak (V̇O_2peak_) is achieved or if the participant is unable to continue for any reason. Ten minutes of rest prior to the test will allow to capture baseline physiological parameters such as heart rate, V̇O_2_, the volume of carbon dioxide expired (V̇CO_2_), pulmonary ventilation during exercise (V̇E), respiratory exchange ratio (RER), excess post-exercise oxygen consumption (EPOC), heart rate recovery, and aerobic and anaerobic recovery thresholds [37] (Supplementary Table 1). After 3 min of warm up at 3 km/h, the speed will be increased to 6 km/h at 0% incline. The treadmill elevation will be increased by 3% every 3 min without changing the speed until volitional fatigue is achieved by the participant. The maximum inclination of the treadmill is set at 21. Rate of perceived exertion (RPE) will be measured at baseline, as well as ~ 30 s before the end of each stage and immediately after the exercise testing. Lactate (mM) and glucose (mM) will be measured from the fingertip capillary blood at baseline and immediately after the exercise testing.

V̇O_2_peak is determined with the following criteria(s): respiratory exchange ratio (RER) ≥ 1.15 and/or blood lactate: ~  + 8 mM above resting values and/or heart rate: ~ 100% of age-predicted HR max/HR reserve and/or RPE of > 17 and/or V̇O_2_ plateau (< 2.1 ml/min/kg in V̇O_2_ despite increase in workload) and/or volitional fatigue [38].

After completion of the exercise, the same physiological variables as in the beginning of the test will continue to be measured during the 30 min of recovery for determining the recovery parameters (Supplementary Table 1).

### Pulse wave analysis and carotid-femoral pulse wave velocity

For analyzing brachial and central aortic blood pressure and arterial stiffness, pulse wave analysis (PWA) and carotid-femoral pulse wave velocity PWV (cfPWV) will be measured by a non-invasive pressure waveform diagnostic tool (SphygmoCor-XCEL, AtCor Medical, Australia) will be used, with the participant in the supine position [39].

For PWA, after a 15-min rest, a brachial pressure cuff will be wrapped around the participant’s arm to obtain the brachial and central aortic blood pressure measurements. For cfPWV a 3-point-subtraction-method is used in this study, when the distance (in mm) from the carotid artery to the suprasternal notch, and from suprasternal notch to femoral artery will be measured with a measuring tape (SECA201, USA) [39]. The carotid and femoral pulse will be assessed simultaneously using a tonometer and a thigh cuff respectively. The arterial pressure wave forms will then be recorded accordingly to obtain cfWPV reading. The following calculation will be used: the distance between suprasternal notch to femoral artery minus the distance between carotid artery to suprasternal notch divided by the pulse transit time. This subtraction method is considered the most accurate non-invasive technique with the obtained cfPWV value closest to the invasive PWV technique [39].

PWA will be followed by cfPWV instantly and repeated twice at the interval of 10 min, and if the difference of the first and second cfPWV measurements is larger than 0.5 m/s, a third measurement will be performed [40]. Higher cfPWV indicates a stiffer aorta [41, 42]. All parameters are listed in Supplementary Table 4.

### Sample collection, analysis and biobanking

Fasted blood will be collected at screening, baseline, 3, 6, and 9 months (Table 2). Buffy coats from the blood samples will be used for DNA methylation. Blood variables during visits 1 to 4 will include the same measurements as the screening visit parameters and insulin (mIU/L), HbA1c (mmol/mol), metabolite concentration (Nightingale Health Plc, Finland), circulating immune and inflammatory markers (Olink, Sweden) (Table 2). Peripheral blood mononuclear cells will be isolated by Ficoll gradient centrifugation (Ficoll-paque, Cytiva, USA) and stored for immunophenotypic analysis (Supplementary Table 2). Any excess blood samples in the form of plasma, serum, frozen buffy coats will be stored for future analysis if the participants consented for it, including participants who do not meet the inclusion criteria after screening.

Fasted saliva samples will be collected from participants in a seated position, where they will passively drool into a collection tube (Isohelix, Kent, UK) pre-filled with guanidine-free, DNA stabilization buffer. Samples will be immediately placed on ice and stored at − 80 °C. Genomic DNA from saliva will similarly be extracted with QIAamp® DNA Mini kits and these samples will be used for genome-wide DNA methylation analysis using the EPIC BeadChip. This will be compared with the results obtained from the blood DNA methylation analysis, aiming to study the association of both tests. The second saliva collection tube will be immediately frozen for future analysis (Supplementary Table 2).

Gut microbiota disturbances have been linked to health outcomes and facilitated by diet and medication [43]. The stool samples for gut microbiota analysis will be collected Zymo Research fecal collection kit and 95% ethanol collection tubes [44–46]. During the baseline, 3, 6, and 9 months visits, participants will receive the stool collection kit with a detailed instruction sheet. The participants will then return the stool container with the samples collected within 24 h. Total nucleic acids are then extracted from each stool sample via the QIAamp PowerFecal Pro DNA Kit® (Qiagen) [44]. Illumina sequencing libraries are prepared from 100 to 500 ng DNA using the TruSeq Nano DNA Library Preparation kit (Illumina) according to the manufacturer’s recommended protocol, with reaction volumes scaled accordingly [47]. Libraries are then sequenced on NovaSeq6000 using PE2 × 150 flowcell to yield 20 million paired end reads. Taxonomic and functional profiles are generated with the bioBakery metabolomic workflow v0.90 [48] (Supplementary Table 2).

### Advanced glycation end-products reader

Glucose reacts with proteins, lipids, and nucleic acids to form first early glycation products and then advanced glycation end-products (AGEs) which are non-reversible and have been implicated in a number of chronic diseases such as diabetes and cardiovascular disease [49–51]. The role of AGEs in aging is still under study, and it has been proposed that AGEs directly or indirectly may contribute to the development of aging-related diseases [51]. AGEs are estimated using the AGE Reader (Diagnoptics Technologies B.V, Netherlands), which has validated against skin biopsies [52], and measures autofluorescence in human skin tissue with ultra-violet (UV) light. AGEs display a characteristic spectrum at 440 nm, which will be displayed in arbitrary units (Supplementary Table 1). The participants will be asked to remove the impurities from the inferior region of the dominant arm with alcohol wipe, and their forearm will be aligned and placed on the device for autofluorescence detection. The measurement will be repeated three times and the average will be calculated.

### Questionnaires

Participants will complete several questionnaires and surveys via Qualtrics (QualtricsXM, SAP, USA), excluding cognitive assessment, which will be administered via MoCA DUO application (MoCA Conginition, Canada). The data collection from questionnaires and surveys includes socio-demographics (Supplementary Table 3), measures of sleep (modified Pittsburgh Sleep Quality Index questionnaire + SATED questionnaire) [53, 54], quality of life: general health by Short Form 36 Health Survey Questionnaire (SF-36) [55], quality of life: intraday health status by EuroQoL 5-level EQ-5D version (EQ-5D-5L) [56], cognitive performance by e-Montreal Cognitive Assessment (MoCA) [57–59], physical activity and sitting by International Physical Activity Questionnaire – Short Form (IPAQ-short) [60], menopause questionnaire [61], nutritional composition and dietary patterns with three-day food record [62], physical activity diary (Supplementary Table 3), and preferences and behaviors [63] (Supplementary Table 3). The pre-intervention data collection includes a pre-trial questionnaire, checking if the participants feel fit to perform the study assessments, if they have fasted and taken their study product, the post-intervention survey includes a feedback form.

### Sample size, randomization, and blinding

For this study, 120 participants will be recruited and equally randomized into Ca-AKG (*n* = 60) or placebo groups (*n* = 60). This number of individuals is based on the previous double-blind randomized study on 40 (20 10 g of ornithine alpha-ketoglutarate and 20 10 g of placebo) hospitalized individuals presenting anorexia and weight loss. An increase in protein and calorie intake was observed after 15 and 30 days of treatment [64]. In a randomized double-blind placebo-controlled trial of older ambulatory patients recovering from acute illnesses [16] patients receiving a different form of ketoglutarate, ornithine oxoglutarate (93 10 g of ornithine oxoglutarate and 93 10 g of placebo), had an improved appetite, gained weight and improved in quality of life. Previous studies focused on different populations and used different forms of AKG, thus, it is not known what the expected effect size of Ca-AKG in healthy individuals is. Including 120 participants was therefore a pragmatic choice.

Participants who meet the recruitment criteria and eligible for the study will be further assigned a new randomization number upon enrolment in the study. The randomization number will be issued in ascending order with no number skipped within a cohort. This will be based on a computer-generated randomization schedule prepared before the enrolment. The randomization will be balanced by using randomly permuted blocks. The random allocation sequence and randomization will be generated by an independent statistician who is not involved in the study and sealed in envelopes. This allocation will be concealed until the end of the study. Investigators and participants will be blinded to study product allocation.

### Statistical methods

To address the hypotheses while considering the randomized control trial design and multiple measurements, the proposed analyses involve several statistical methods. Initial descriptive analyses (e.g., *t*-test, analysis of variance, Mann-Whitney test, Kruskal-Wallis test, Chi-square test, and Fisher’s exact test) will be performed. For continuous outcomes, linear regression models will be constructed to compute mean difference and 95% confidence interval (CI); similarly, binary logistic regression models will be constructed to compute odds ratios and 95% CI for results with binary outcomes. Generalized linear model, generalized estimating equations, generalized linear mixed model, time-to-event and receiver operating characteristic curve (ROC) analysis will be used to analyze the outcomes. Data will be analyzed by statistical analysis software (RStudio, Stata and SPSS) and all statistical tests will be conducted at 5% level of significance.

### Safety assessments

Ca-AKG is Generally Recognized As Safe (GRAS), thus, no adverse events are expected to be related to the effect of Ca-AKG. However, documentation of all adverse events reported by participants will be recorded. All serious adverse events will be reported to the ethics committee (NUS-IRB) and to Health Science Authority of Singapore within 24 h. In addition, to ensure safety, liver function, kidney function, and complete blood test will be done at 3, 6, and 9 months.

## Discussion

Geroscience aims to investigate the molecular and cellular mechanisms that contribute to aging. A demonstration that a supplement can modify the mechanisms that were identified as pillars of aging are the first step in moving towards targeting aging at the molecular and cellular level in humans. This study is the double-blind randomized placebo-controlled study investigating the potential of Ca-Alpha-Ketoglutarate supplementation to reduce DNA methylation age and the effect on clinical and biological outcomes. In addition, the screening phase will inform on the proportion of 40–60-year-old individuals displaying an older biological DNA methylation age based on the mean of four DNA methylation clocks, Hannum, Horvath’s, GrimAge and PhenoAge. This information is important to assess the feasibility of recruiting subjects based on their DNA methylation age in the absence of any chronic diseases.

Future studies focusing on the effect of Ca-AKG on aging could investigate combinations with Ca-AKG and lifestyle and pharmaceutical interventions. It is important for translation into the clinic to understand what recommendations to give to people when prescribing Ca-AKG. For example, it has been previously reported that AKG is an important mediator of resistance-exercise metabolic effects [65]. Thus, investigating the effect of exercise when combined with Ca-AKG compared to exercise alone on muscle health and metabolism, might shed light whether some individuals would need to add Ca-AKG to their exercise routine. Another important study would need to address how long the effect of Ca-AKG would last. A study with longer follow up would be able to show if the changes induced by Ca-AKG are sustained for longer. This would inform if people need to take AKG continuedly or periodically. This study is the first to investigate the effect of sustained-release Ca-AKG on DNA methylation in DNAm-older individuals.

### Supplementary Information

Below is the link to the electronic supplementary material.

Supplementary file1 (DOCX 61.9 KB)
