# Supplementary material for: Alpha-ketoglutarate supplementation and BiologicaL agE in middle-aged adults (ABLE)—intervention study protocol
Source: GeroScience. 2023 May 23;45(5):2897–907. doi: 10.1007/s11357-023-00813-6 (PMC10643463; doi:10.1007/s11357-023-00813-6)
Supplement: Supplementary file 1 — Supplementary file1 (DOCX 61.9 KB) [file 11357_2023_813_MOESM1_ESM.docx]

Supplementary material

Supplementary Table 1. Body composition, functional tests, cardiopulmonary exercise test (CPET), activity tracking

| Test | Parameters, units |
| --- | --- |
| DXA | Bone Mineral Density, g/cm^2^  Fat mass, kg  Fat-free mass, kg  Left Arm Area, cm^2^  Left Arm Bone Mineral Content, g  Left Arm Bone Mineral Density, g/cm^2^  Right Arm Area, cm^2^  Right Arm Bone Mineral Content, g  Right Arm Bone Mineral Density, g/cm^2^  Left Rib Area, cm^2^  Left Rib Bone Mineral Content, g  Left Rib Bone Mineral Density, g/cm^2^  Right Rib Area, cm^2^  Right Rib Bone Mineral Content, g  Right Rib Bone Mineral Density, g/cm^2^  Thorax Spine Area, cm^2^  Thorax Spine Bone Mineral Content, g  Thorax Spine Bone Mineral Density, g/cm^2^  Lumbar Spine Area, cm^2^  Lumbar Spine Bone Mineral Content, g  Lumbar Spine Bone Mineral Density, g/cm^2^  Pelvis Area, cm^2^  Pelvis Bone Mineral Content, g  Pelvis Bone Mineral Density, g/cm^2^  Left Leg Area, cm^2^  Left Leg Bone Mineral Content, g  Left Leg Bone Mineral Density, g/cm^2^  Right Leg Area, cm^2^  Right Leg Bone Mineral Content, g  Right Leg Bone Mineral Density, g/cm^2^  Subtotal Area, cm^2^  Subtotal Bone Mineral Content, g  Subtotal Bone Mineral Density, g/cm^2^  Head Area, cm^2^  Head Bone Mineral Content, g  Head Bone Mineral Density, g/cm^2^  Total Area, cm^2^  Total Bone Mineral Content, g  Total Bone Mineral Density, g/cm^2^  Abdominal Fat Mass, g  Abdominal Lean Mass + Bone Mineral Content, g  Abdominal Total Mass, g  Abdominal Fat Percentage, %  Hip/Buttock Fat Mass, g  Hip/Buttock Lean Mass + Bone Mineral Content, g  Hip/Buttock Total Mass, g  Hip/Buttock Fat Percentage, %  Total Body Fat Percentage, %  Estimated Visceral Adipose Tissue Mass, g  Estimated Visceral Adipose Tissue Volume, cm3  Estimated Visceral Adipose Tissue Area, cm^2^  Left Arm Bone Mineral Content, g  Left Arm Fat Mass, g  Left Arm Lean Mass, g  Left Arm Lean Bone Mineral Content, g  Left Arm Total Mass, g  Left Arm Fat Mass, g  Right Arm Bone Mineral Content, g  Right Arm Fat Mass, g  Right Arm Lean Mass, g  Right Arm Lean Bone Mineral Content, g  Right Arm Total Mass, g  Right Arm Fat Mass, g  Trunk Bone Mineral Content, g  Trunk Fat Mass, g  Trunk Lean Mass, g  Trunk Lean Bone Mineral Content, g  Trunk Total Mass, g  Trunk Fat Mass, g  Left Leg Bone Mineral Content, g  Left Leg Fat Mass, g  Left Leg Lean Mass, g  Left Leg Lean Bone Mineral Content, g  Left Leg Total Mass, g  Left Leg Fat, g  Right Leg Bone Mineral Mass, g  Right Leg Fat Mass, g  Right Leg Lean Mass, g  Right Leg Lean Bone Mineral Content, g  Right Leg Total Mass, g  Right Leg Fat Mass, g  Subtotal Bone Mineral Content, g  Subtotal Fat Mass, g  Subtotal Lean Mass, g  Subtotal Lean Bone Mineral Content, g  Subtotal Total Mass, g  Subtotal Fat Mass, g  Head Bone Mineral Content, g  Head Fat Mass, g  Head Lean Mass, g  Head Lean Bone Mineral Content, g  Head Total Mass, g  Head Fat Mass, g  Total Bone Mineral Content, g  Total Fat Mass, g  Total Lean Mass, g  Total Lean Bone Mineral Content, g  Total Mass, g  Total Fat Percentage, %  Neck Area, cm^2^  Neck Bone Mineral Content, g  Neck Bone Mineral Density, g/cm^2^  Total Area, cm^2^  Total Bone Mineral Content, g  L1 Area, cm^2^  L1 Bone Mineral Density, g/cm^2^  L2 Area, cm^2^  L2 Bone Mineral Density, g/cm^2^  L3 Area, cm^2^  L3 Bone Mineral Density, g/cm^2^  L4 Area, cm^2^  L4 Bone Mineral Density, g/cm^2^  Total Area A, cm^2^  Total Bone Mineral Content A, g  Total Bone Mineral Density A, g/cm^2^ |
| Handgrip strength | Right hand: strength, kg |
|  | Left hand: strength, kg |
| Leg extension strength | 8-Repetition Maximal, kg |
| Cardiopulmonary exercise test (CPET) | Volume of Oxygen consumption (V̇O_2_), L/min  Volume of Oxygen consumption per kg body mass (V̇O_2_/kg), L/min/kg  Peak V̇O_2_ (VO_2peak_), L/min  Volume of Carbon Dioxide production (V̇CO_2_)  Volume of carbon dioxide production per kg body mass (V̇CO_2_/kg), L/min/kg  Volume of expired air per min/Minute ventilation (V̇E), L/min  Breathing frequency, breath/min  Respiratory Exchange Ratio, ratio  Ventilatory equivalent for oxygen (V̇E/ V̇O_2_), ratio  Ventilatory equivalent for carbon dioxide (V̇E/ V̇CO_2_), ratio  Heart rate (HR), beats/min (bpm)  End tidal partial pressure of oxygen (PetO2), mmHg  End tidal partial pressure of carbon dioxide (PetCO2), mmHg  Aerobic threshold  Anaerobic threshold  Heart rate recovery (HRR), beats/min (bpm)  Excess post exercise oxygen consumption:   - Magnitude - Duration, mins:secs - Area under curve. au   Rate of Perceived Exertion, scale from 6 no exertion –20 maximal exertion [1]  Blood lactate, mM  Blood glucose, mM |
| Physical activity motion tracking | 3-axis (Forward, side and vertical) accelerations, m/s ^2^ |
| AGE reader | Skin autofluorescence, au |

Supplementary Table 2. Assessments and parameters in biological samples

| Sample | Assessments | Parameters |
| --- | --- | --- |
| Blood | DNA methylation: EPIC chip | DNAm age, years |
|  | Fasting haematological panel | white blood cell count (WBC) |
|  |  | red blood cell count (RBC) |
|  |  | hematocrit (Hct) |
|  |  | hemoglobin (Hbg) |
|  |  | platelet count/volume |
|  | Fasting lipid panel | total cholesterol, mmol/l |
|  |  | HDL cholesterol, mmol/l |
|  |  | LDL cholesterol, mmol/l |
|  |  | Triglycerides, mmol/l |
|  | Fasting glucose | glucose, mg/dl |
|  | Fasting protein panel | albumin, g/dl |
|  |  | globulin, g/dl |
|  | Renal function test | serum creatinine, mg/dl |
|  |  | blood urea nitrogen (BUN), mg/dl |
|  | Insulin & HbA1C | insulin, mIU/l |
|  |  | HbA1C, mmol/mol |
|  | Metabolite concentrations | alanine, mmol/l |
|  |  | glutamine, mmol/l |
|  |  | glycine, mmol/l |
|  |  | histidine, mmol/l |
|  |  | isoleucine, mmol/l |
|  |  | leucine, mmol/l |
|  |  | valine, mmol/l |
|  |  | phenylalanine, mmol/l |
|  |  | tyrosine, mmol/l |
|  |  | apolipoproteins A1/B, g/l |
|  |  | LDL cholesterol, mmol/l |
|  |  | HDL cholesterol, mmol/l |
|  |  | remnant cholesterol, mmol/l |
|  |  | total cholesterol, mmol/l |
|  |  | VLDL cholesterol, mmol/l |
|  |  | total esterified cholesterol, mmol/l |
|  |  | cholesterol esters (in HDL, LDL, VLDL), mmol/l |
|  |  | docosahexaenoic acid, mmol/l |
|  |  | linoleic acid, mmol/l |
|  |  | monounsaturated fatty acids, mmol/l |
|  |  | omega-3 fatty acids, mmol/l |
|  |  | omega-6 fatty acids, mmol/l |
|  |  | polyunsaturated fatty acids, mmol/l |
|  |  | saturated fatty acids, mmol/l |
|  |  | total fatty acids, mmol/l |
|  |  | citrate, mmol/l |
|  |  | glucose, mmol/l |
|  |  | glycerol, mmol/l |
|  |  | lactate, mmol/l |
|  |  | pyruvate, mmol/l |
|  |  | glycoprotein acetyls, mmol/l |
|  |  | 3-Hydroxybutyrate, mmol/l |
|  |  | acetone, mmol/l |
|  |  | acetoacetate, mmol/l |
|  |  | acetate, mmol/l |
|  |  | phosphatidylcholines, mmol/l |
|  |  | phosphoglycerides, mmol/l |
|  |  | sphingomyelins, mmol/l |
|  |  | total choline, mmol/l |
|  | Biobanking | future analysis |
| Saliva | DNA methylation | DNAm, years |
|  | Biobanking | Future analysis |
| Stool | Metagenomic sequencing | Bacterial species and pathways |
|  | Short Chain Fatty Acids | Acetic acid (C2:0) |
|  |  | Propionic acid (C3:0) |
|  |  | Butyric acid (C4:0) |
|  |  | Isobutyric acid (C4:0i) |
|  |  | Valeric acid (C5:0) |
|  |  | Isovaleric acid (C5:0i) |
|  |  | Hexanoic acid (C6:0) |

Supplementary table 3: Questionnaires assessments and outcomes

| **Questionnaire** | **Assessment** | **Outcome** | **References** |
| --- | --- | --- | --- |
| Socio-demographic Survey | Demographic information | Marital status  Living partner  Accommodation  Residency status  Country of born and residence  Races  Education level  Smoking habits  Alcoholic habits  Family members  Work status  Occupation | N.A. |
|  |  |  |  |
| Sleep Questionnaire (modified Pittsburgh Sleep Quality Index questionnaire + SATED questionnaire) | Sleep Quality    Sleep Health in 5 domains:  satisfaction  alertness  timing  efficiency  duration | modified Pittsburgh Sleep Quality Index questionnaire  Quantitative:   1. sleep duration [min score = 0 (better); max score = 3 (worse)] 2. sleep quality score [min score = 0 (better); max score = 3 (worse)]     SATED questionnaire  Quantitative: sleep health score  [min. score = 0 (poor sleep health); max. score = 10 (good sleep health)] | [2, 3] |
|  |  |  |  |
| Short Form 36 Health Survey Questionnaire (SF-36) | Health-related quality of life in eight health domains:  physical functioning  bodily pain  role limitations due to physical health problems,  role limitations due to personal or emotional problems  emotional well-being,  social functioning  energy/fatigue  general health perceptions | Quantitative: Physical component summary (PCS); Mental component summary (MCS) | [4] |
|  |  |  |  |
| EuroQoL 5-level EQ-5D version (EQ-5D-5L) | Intraday health status in five dimensions:  mobility  self-care  usual activities  pain/discomfort  anxiety/depression    Overall self-rated health status: vertical visual analogue scale (EQ VAS) scores | Quantitative health status:   1. descriptive score in each dimension [min. score = 1 (no problem); max. score = 5 (extreme problem)] 2. health state: 5-digit code (indicates score for each dimension)     Qualitative health status in each dimension:  LEVEL 1: indicating no problem  LEVEL 2: indicating slight problems  LEVEL 3: indicating moderate problems  LEVEL 4: indicating severe problems  LEVEL 5: indicating unable to/extreme problems    Quantitative: EQ VAS scores [min. score = 0 (worst); max. score = 100 (best)] | [5, 6] |
|  |  |  |  |
| Montreal Cognitive Assessment (MoCA) | Cognitive performance | Quantitative: MoCA total score (__/30)    Qualitative: normal (≥26/30) | [7-9] |
|  |  |  |  |
| International Physical Activity Questionnaire – Short Form (IPAQ-short) | Subjective physical activity level | Quantitative: Total physical activity (MET-minutes/week)    Qualitative: inactive; minimally active; or HEPA active | [10] |
|  |  |  |  |
| Menopause Questionnaire | Menopause information    Mood symptoms during peri-menopause and post-menopause | Quantitative: descriptive score in each mood symptom (not at all, mild, moderate, or severe) | [11] |
|  |  |  |  |
| 3-day food diary | Nutritional composition of diet, dietary patterns | Quantitative: macro-/ micro-nutrients    Qualitative: dietary patterns | N.A. |
|  |  |  |  |
| 7-day physical activity diary | The diary/record of participants’ physical activity information for 7 days while wearing ActiGraph and each time when the ActiGraph is removed | Allow the access, and for the comparison of the 7-day physical activity information with ActiGraph’s activity tracking data | N.A. |
|  |  |  |  |
| Global Preferences Survey | preference(s) or economic behavioural change(s) before/during/after the intervention study:  Risk and Time preferences  Expectations about stock market development and health status  Social preference: positive and negative reciprocity, altruism and trust | Qualitative:   1. the amount for the risk and the time preferences (10-point Likert scale) 2. Expectations about stock market development and health status (percentage & yes or no response) 3. Social preferences (10-point Likert scale & Hypothetical subjective response)     Quantitative: the amount for the risk and the time preferences are assessed via staircase method | [12] |

Supplementary Table 4. Pulse wave analysis and Pulse wave velocity

| Text | Assessments | Parameters, units |
| --- | --- | --- |
| Pulse wave analysis (PWA) | Brachial systolic and diastolic blood pressure | Resting blood pressure, mmHg |
|  |  |  |
|  | Central aortic systolic and diastolic blood pressure | Aortic systolic and diastolic blood pressure (SP and DP), mmHg |
|  |  |  |
|  | The difference between the maximum and minimum of the central pressure waveform (the height of the aortic pressure waveform or SP minus DP) | Aortic pulse pressure (PP), mmHg |
|  |  |  |
|  | The average aortic pressure in a pulse | Aortic mean arterial pressure (MAP), mmHg |
|  |  |  |
|  | Average heart rate in beats per minute during the measurement | Aortic heart rate (HR), beats per minutes (bpm) |
|  |  |  |
|  | The difference between two pressure peaks during systole. It is a measure of wave reflected back from lower body | Augmentation pressure (AP), mmHg |
|  |  |  |
|  | The ratio of AP to PP in percentage | Augmentation Index (AIx), % |
|  |  |  |
|  | AIx normalized to a heart rate of 75 bpm | Augmentation Index 75 (AIx75), % |
|  |  |  |
| Pulse wave velocity (PWV): subtraction method | Carotid-femoral pulse wave velocity in metres/seconds, calculated as the distance in metre divided by pulse transit time in millisecond. PWV is averaged over the reading. | Pulse wave velocity (PWV), m/s |

Supplementary Table 5. Ingredients of Ca-AKG and placebo

| Treatment | Ingredients | Amount, mg |
| --- | --- | --- |
| Ca-AKG | Ca-alpha-ketoglutarate | 500 |
| Placebo | Micro crystal cellulose  Stearic Acid  Veg Wax  Magnesium Stearate  Silica  Total tab weight | 500  160  7  0.008  0.003  738 |

References

1. Borg, G., *Borg's Perceived Exertion And Pain Scales*. 1998.

2. Buysse, D.J., *Sleep health: can we define it? Does it matter?* Sleep, 2014. **37**(1): p. 9-17.

3. Buysse, D.J., et al., *The Pittsburgh Sleep Quality Index: a new instrument for psychiatric practice and research.* Psychiatry research, 1989. **28**(2): p. 193-213.

4. Ware Jr, J.E., *SF-36 health survey update.* Spine, 2000. **25**(24): p. 3130-3139.

5. Herdman, M., et al., *Development and preliminary testing of the new five-level version of EQ-5D (EQ-5D-5L).* Quality of life research, 2011. **20**(10): p. 1727-1736.

6. Foundation, E.R. *EQ-5D-5L User Guide: Basic information on how to use the EQ-5D-5L instrument*. 2021 [cited 2022 16th August]; Available from: <https://euroqol.org/publications/user-guides/>.

7. Julayanont, P. and Z.S. Nasreddine, *Montreal Cognitive Assessment (MoCA): concept and clinical review*, in *Cognitive screening instruments*. 2017, Springer. p. 139-195.

8. Nasreddine, Z.S. *Montreal Cognitive Assessment (MoCA) Administration and Scoring Instructions*. 2010 [cited 2022 23th June]; Available from: <https://www.smchealth.org/sites/main/files/file-attachments/moca-instructions-english_2010.pdf>.

9. Nasreddine, Z.S., et al., *The Montreal Cognitive Assessment, MoCA: a brief screening tool for mild cognitive impairment.* Journal of the American Geriatrics Society, 2005. **53**(4): p. 695-699.

10. Committee, I.R., *Guidelines for data processing and analysis of the International Physical Activity Questionnaire (IPAQ)-short and long forms.* <http://www>. ipaq. ki. se/scoring. pdf, 2005.

11. Health, T.J.H.C.f.W.s.R.M. *Perimenopausal Evaluation*. n.d. [cited 2022 16th August]; Available from: <https://www.hopkinsmedicine.org/psychiatry/specialty_areas/moods/patient_information/Perimenopausal_Evaluation>.

12. Falk, A., et al., *Global evidence on economic preferences.* The Quarterly Journal of Economics, 2018. **133**(4): p. 1645-1692.
